# A novel sample preparation strategy for shotgun lipidomics of phospholipids employing multilamellar vesicles

**DOI:** 10.1007/s00216-018-1113-8

**Published:** 2018-05-08

**Authors:** Melissa Frick, Tommy Hofmann, Caroline Haupt, Carla Schmidt

**Affiliations:** Interdisciplinary Research Center HALOmem, Charles Tanford Protein Center, Martin Luther University Halle-Wittenberg, Kurt-Mothes-Str. 3a, 06120 Halle (Saale), Germany

**Keywords:** Lipidomics, Phospholipids, Mass spectrometry, Liposomes

## Abstract

The identification of lipids in biological samples is gaining importance. The advent of mass spectrometry-based lipidomics accelerated the field allowing nowadays for identification and quantification of complete lipidomes. However, due to solubility difficulties and varying properties of different lipid classes, sample preparation for lipidomics is still an issue. Of the many lipid classes, phospholipids are the major components of biological membranes. In solution, they spontaneously form lipid vesicles of various structures such as liposomes. They are therefore often used as membrane mimics when studying biological membranes and membrane proteins. Here, we present a novel sample preparation strategy for shotgun lipidomics employing liposomes prepared from lipid standards or lipid mixtures allowing the analysis of phospholipids directly from lipid bilayers. We validated our strategy for lipid identification by tandem mass spectrometry in positive or negative ion mode using different phospholipid species from various classes. We further tested our strategy for relative quantification by mixing different ratios of phospholipid species as well as determining the distribution of lipid species in a natural lipid extract.

**Electronic supplementary material:**

The online version of this article (10.1007/s00216-018-1113-8) contains supplementary material, which is available to authorized users.

**Graphical abstract:**
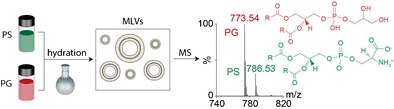
ᅟ

## Introduction

Lipids are diverse biomolecules that are involved in different biological processes such as energy storage, signalling and membrane trafficking as well as acting as structural components in biological membranes. Glycerophospholipids (also called phospholipids) are, due to their amphiphilic nature, structured in an aqueous environment; they form micelles or lipid bilayers such as liposomes or planar membranes, facing the hydrophilic head group towards the aqueous solvent and burying the hydrophobic hydrocarbon chain in the membrane. Based on their polar head group, they are divided into subclasses including phosphatidylcholine (PC), phosphatidylethanolamine, phosphatidylserine (PS) or phosphatidylinositol (PI). PIs and their phosphorylated derivatives are rather underrepresented in biological membranes and act as signalling molecules [1, 2].

The use of high-resolution and sensitive mass spectrometers increased our knowledge and allowed qualitative and quantitative descriptions of entire lipidomes [3, 4]. In mass spectrometry (MS)-based lipidomics, lipids are identified by their intact masses and by dissociation into specific fragments [5]. For MS analysis, lipids are usually extracted from cellular tissue or extracts following classical extraction protocols [6, 7] or further developed protocols [8]. However, the use of organic solvents implicates the risk of precipitation of co-purified contaminating compounds such as salt or components of the biological matrix [8].

We introduce a novel sample preparation strategy based on preparation of liposomes allowing the analysis of lipids from membrane-like structures. For this, lipids dissolved in organic solvents are mixed in the desired ratio and a lipid film is generated by evaporation of the solvent followed by hydration in ammonium acetate, a volatile buffer commonly used in MS analysis of biomolecules. During hydration, lipid sheets detach from the lipid film and multilamellar vesicles (MLVs) are formed [9]. We show that these MLVs can be employed during shotgun lipidomics for both determination of intact lipid masses and dissociation into specific fragments. Varying lipid contents are reflected in correct intensity ratios in the mass spectra obtained from liposomes representing promising future quantitative applications. Importantly, we show that liposomes can be completely dissociated during transfer in the gas phase of the mass spectrometer making them promising vehicles for analysis of insoluble, membrane-bound biomolecules.

## Materials and methods

### Lipid standards

1,2-Dioleoyl-sn-glycero-3-phosphocholine (PC 18:1/18:1), 1,2-dioleoyl-sn-glycero-3-phospho-l-serine (PS 18:1/18:1), 1,2-dioleoyl-sn-glycero-3-phospho-(1′-rac-glycerol) (PG 18:1/18:1), 1-palmitoyl-2-linoleoyl-sn-gylcero-3-phospho-(1′-rac-glycerol) (PG 16:0/18:2) and l-α-phosphatidylinositol (Soy-PI) were purchased from Avanti Polar Lipids Inc. (Alabaster, AL, USA). The lipid shorthand nomenclature proposed by Liebisch et al. [10] was used throughout the manuscript.

### Preparation of liposomes

For liposome preparation, lipids were dissolved in chloroform and mixed in different proportions. The solvent was then evaporated using a rotary evaporator. To obtain MLVs, the lipid film was hydrated with 200 mM ammonium acetate by rotation in a rotary evaporator at room temperature for approx. 1 h without evaporation. MLVs were stored at 4 °C until use. Unilamellar vesicles (ULVs) were prepared from MLVs by 21 strokes through a 0.2-μm polycarbonate membrane (Millipore Inc., Bedford, MA, USA) using a mini-extruder.

### Dynamic light scattering

The average potential diameters of MLVs and ULVs were measured by dynamic light scattering using a Zetasizer Nano (Malvern Instruments, Worcestershire, UK). The average values of liposome sizes were determined from liposomal size distribution histograms.

### Shotgun lipidomics

Liposomes were analysed by direct infusion electrospray ionisation (ESI) on a Q Exactive Plus Hybrid Quadrupole-Orbitrap Mass Spectrometer (Thermo Fisher Scientific). For this, 2–4 μl of sample was loaded into a borosilicate offline emitter coated with gold/palladium (Thermo Fisher Scientific). ESI source settings were electrospray capillary voltage, 1.7–4.0 kV; capillary temperature, 250 °C; resolution, 70.000; and RF-lens level, 50. Spectra were recorded in negative or positive ion mode. The MS scan range was 500–1000 *m*/*z*. Precursors with a selection window of 2 *m*/*z* were selected manually for higher-energy collisional dissociation (HCD). HCD energy varied from 10 to 40 (N)CE. For comparison, lipids were dissolved in 80% (*v*/*v*) methanol/20% (*v*/*v*) water and analysed as described.

### Data analysis

Fragment spectra of lipids were assigned manually. Relative quantification of PG 18:1/18:1 and PG 16:0/18:2 was performed by calculating intensity ratios from the total intensities of both lipid species, i.e. the sum of intensities for all peaks of their isotopic pattern. Relative intensities for PI lipids were calculated from theoretical isotope patterns for each PI species.

## Results

### Multilamellar vesicles for shotgun lipidomics

Liposomes are popular tools in biophysics and structural biology to mimic natural membrane environments. However, due to size and heterogeneity, their application to mass spectrometry-based approaches is limited. We envision that, when using denaturing nano-ESI, intact liposomes can be fully dissociated during transfer into the gas phase of a mass spectrometer yielding monomeric lipid species and therefore allowing the analysis of their lipid components by MS. To test this hypothesis, we first prepared liposomes from PC 18:1/18:1, PG 18:1/18:1 and PS 18:1/18:1 lipid standards in a 1:1:1 M ratio (see Electronic Supplementary Material (ESM) Fig. S1). We chose these lipid species because (i) they are commonly used for liposome preparation, (ii) they have neutral (PC) and negative (PG and PS) net charges at pH 7.4, (iii) they yield characteristic fragment ions in positive (PC) or negative (PG and PS) ion modes and (iv) they have similar gel-liquid crystalline transition temperatures allowing their hydration at room temperature. Following hydration in ammonium acetate, the first liposome products are MLVs with undefined diameter as observed by dynamic light scattering (see ESM Fig. S2). We directly analysed these MLVs by nanoESI-MS using a Q Exactive plus mass spectrometer (see “Materials and Methods” for details). In negative ion mode, two lipid species corresponding to PG 18:1/18:1 (*m/z* 773.53) and PS 18:1/18:1 (*m/z* 786.53) were observed. The spectrum acquired in positive ion mode shows PC 18:1/18:1 (*m/z* 786.60) (see ESM Fig. S1).

**Fig. 1:**
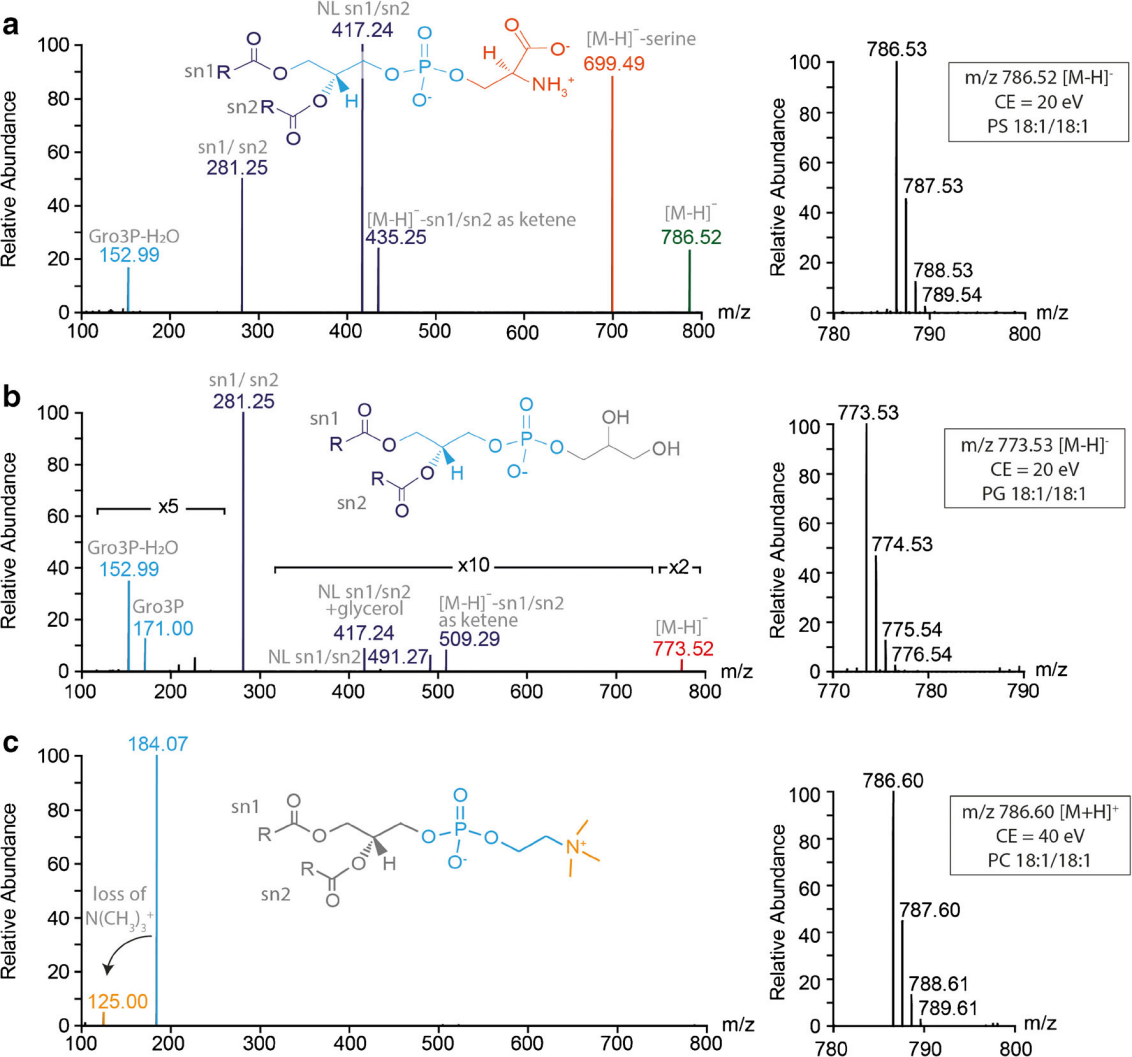
Lipid identification by tandem MS. MSMS spectra (lhs) of PS 18:1/18:1 (*m/z* 786.53) (**a**), PG 18:1/18:1 (*m/z* 773.53) (**b**) and PC 18:1/18:1 (*m/z* 786.60) (**c**) are shown. For each lipid, the precursor isotope pattern is shown (rhs). Fragments specific to lipid head groups and fatty acyl chains are highlighted. Fragment ions are labelled: NL, neutral loss; Gro3P, glycerol-3-phosphate. Of note, *m*/*z* regions of low intensity containing fragment ions were magnified (× 2, × 5, × 10)

The presence of PG, PS and PC standard lipids was confirmed by tandem MS in negative (PG and PS) or positive (PC) ion modes. Tandem mass spectra of PG 18:1/18:1 and PS 18:1/18:1 showed several fragment ions confirming their head group and fatty acyl chains (Fig. 1a, b). Similar to previous studies [11], the tandem mass spectrum of PC 18:1/18:1 showed only fragment ions corresponding to the PC head group (*m*/*z* 184.07 and 125.00) in positive ion mode (Fig. 1c).

To test whether the formation of MLVs is sufficient for MS analysis or if further purification by extrusion yielding ULVs is preferred, we also prepared ULVs using the same lipid proportions (see above, see ESM Fig. S1a). Dynamic light scattering confirmed a defined average diameter of ULVs when compared with MLVs (see ESM Fig. S2). Lipid species observed in negative (see ESM Fig. S1b) and positive (see ESM Fig. S1c) ion mode spectra are identical with those obtained directly from MLVs. Intensity ratios of PG 18:1/18:1 versus PS 18:1/18:1 are comparable for MLVs and ULVs suggesting that ionisation is efficient in both preparations. As preparation of ULVs requires an additional preparation step, the use of MLVs is preferred for lipidomic sample preparation unless the use of ULVs is required for other reasons.

To test whether mass spectra of lipids obtained from liposomes differ from those obtained from lipids dissolved in organic solvents, we analysed the same lipid mixture directly from organic solvent. The mass spectra acquired in negative and positive ion mode are comparable with those obtained from MLVs and ULVs (see ESM Fig. S1b and c); however, some background signals were observed in positive ion mode (see ESM Fig. S1c).

### Relative quantification of different lipid species of the same lipid class

Although equal amounts of PG 18:1/18:1 and PS 18:1/18:1 were used during liposome preparation, MS spectra in negative ion mode revealed different intensities for these two lipids suggesting different ionisation efficiency for different phospholipid classes. To test if a relative comparison of different lipid species of the same phospholipid class is possible using our sample preparation strategy, we mixed PG 18:1/18:1 and PG 16:0/18:2 in varying ratios and prepared MLVs as described (see “Materials and methods” and ESM Fig. S1a for details). Tandem MS confirmed the fatty acyl side chain composition of both species (see Fig. 1b and ESM Fig. S3). The obtained mass spectra showed PG 18:1/18:1 (*m/z* 773.53) as well as PG 16:0/18:2 at *m/z* 745.51 at varying intensities according to their mixing ratio (see ESM Fig. S4).

**Fig. 2:**
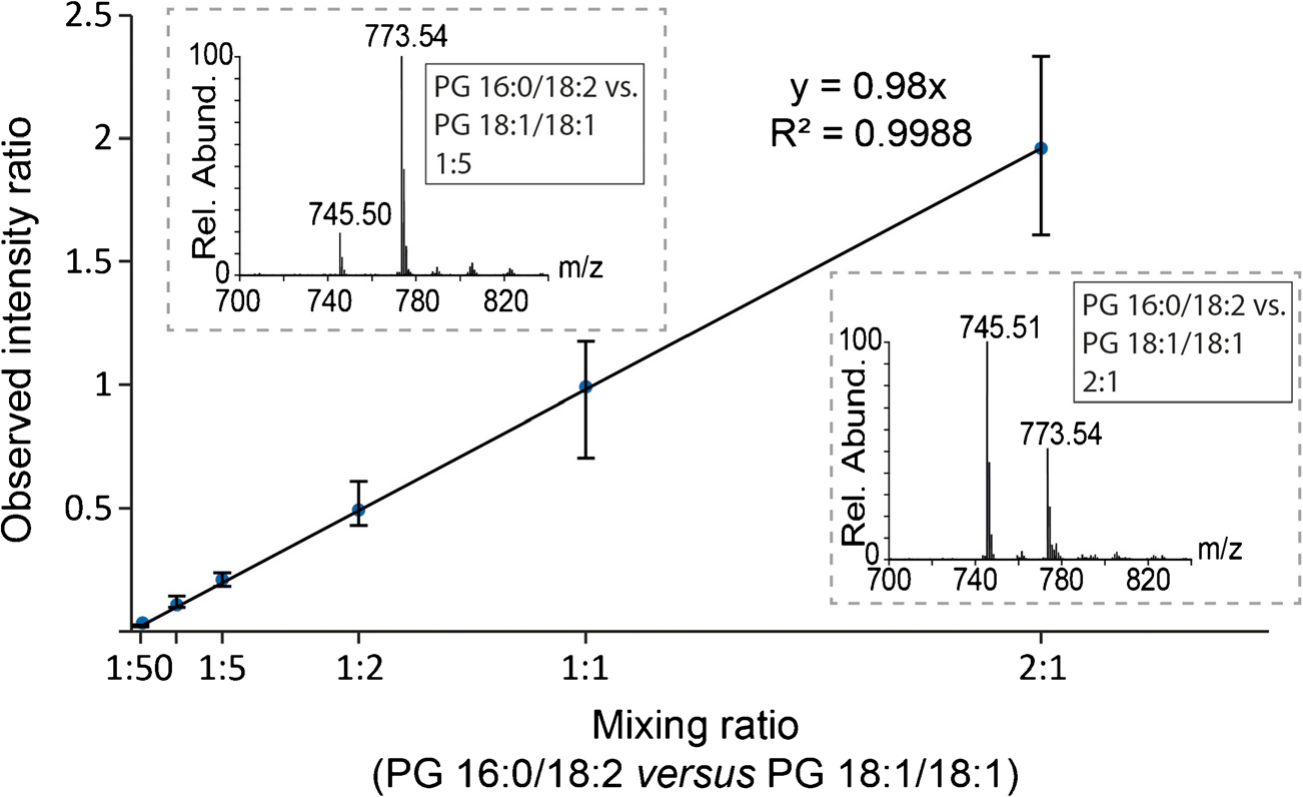
Relative quantification of PG 16:0/18:2 versus PG 18:1/18:1 in different ratios (1:50, 1:10, 1:5, 1:2, 1:1, 2:1). The average mixing ratio of three liposome preparations was calculated (see ESM Table S1). Error bars show the lowest and highest observed ratio. The insets show two example spectra: mixing ratios 1:5 (lhs) and 2:1 (rhs)

To test linearity of this quantification, we prepared MLVs from PG 16:0/18:2 and PG 18:1/18:1 in 2:1, 1:1, 1:2, 1:5, 1:10 and 1:50 ratios and analysed them by denaturing nanoESI-MS. From these MS spectra, we calculated intensity ratios corresponding to *m/z* 745.51 versus *m/z* 773.54 and found that the observed intensity ratios correspond well with the mixing ratio during liposome preparation. Relative quantification of lipid species of the same lipid class is therefore possible and linear from a 2:1 ratio down to 1:50 (Fig. 2). We conclude that relative intensities in MLVs prepared from lipid mixtures or extracts reflect the initial relative abundancies of the lipids. Comparing the intensities of lipid species across the same lipid class, including the theoretical isotope pattern which overlaps for the various species, will therefore provide information on the lipid species present as well as their abundance in the lipid mixture.

### Low abundant lipid species can be identified from multilamellar vesicles

In our sample preparation strategy, we used MLVs at concentrations of 0.5 mM total lipid content. To test whether lower concentrations of lipids are acceptable, we diluted MLVs down to 0.5 μM total lipid concentration (i.e. 1000-fold). At this concentration, we could still observe PG 18:1/18:1 in MS and tandem MS spectra (see ESM Fig. S5) confirming that lower concentrations can also be used for MLV preparation.

**Fig. 3:**
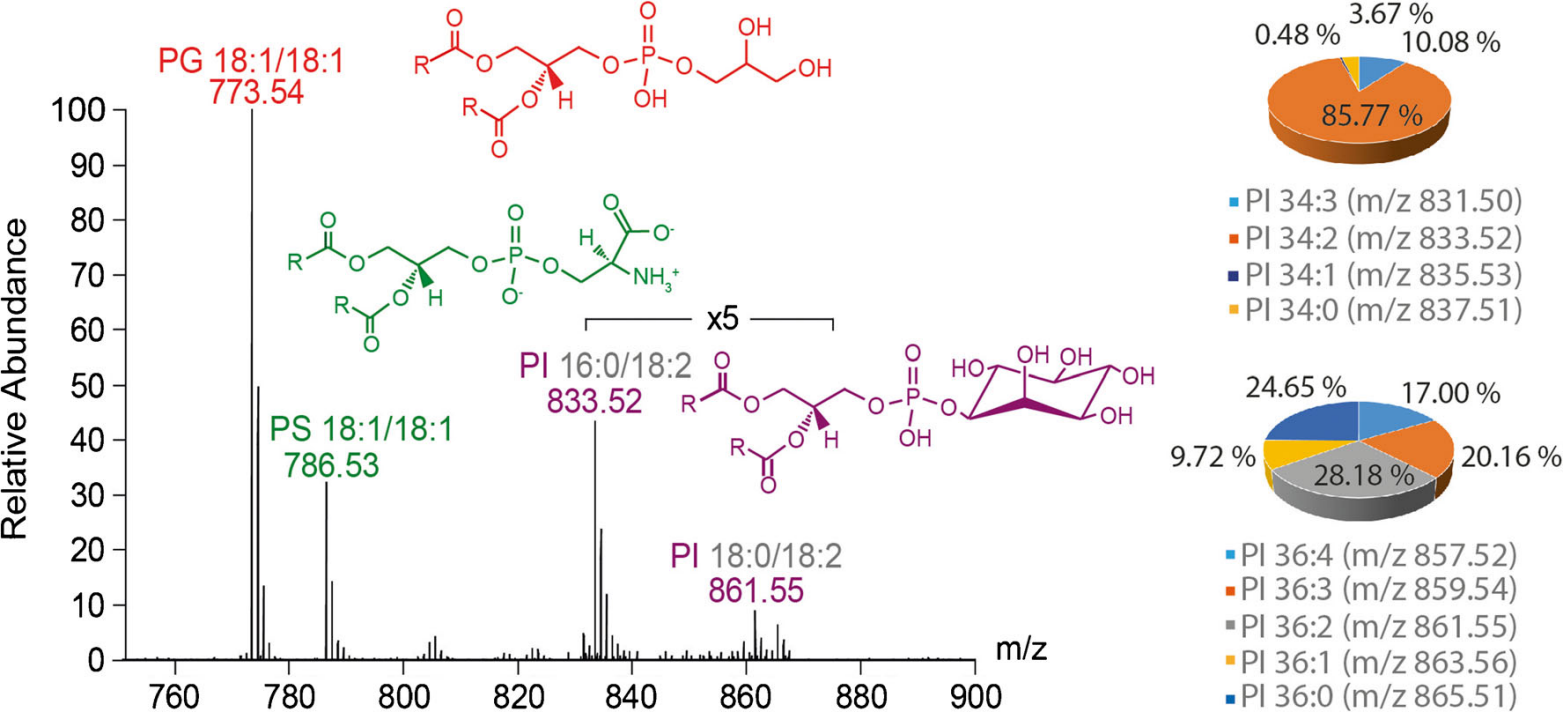
Mass spectrum of mixed MLVs containing PI. PG 18:1/18:1 (red), PS 18:1/18:1 (green) and PI (purple) were mixed in a ratio of 1:1:0.3 (PG:PS:PI). Abundant PI isomers (i.e. PI 16:0/18:2 and PI 18:0/18:2) were identified by tandem MS. Relative quantification of PI isomers was performed by making use of their theoretical isotope pattern. The abundance of PI isomers is shown in pie charts

In natural membranes, PIs are present at lower concentration (approx. 10% of the total lipid content). To mimic their natural abundance, we prepared MLVs using PG, PS and Soy-PI with 1:1:0.3 M ratio (i.e. PI equals approx. 13% of the total lipid content) and analysed them as described. In these mass spectra, we identified PG 18:1/18:1, PS 18:1/18:1 as well as two peak distributions, > 830 *m/z* and > 850 *m/z*, suggesting that more than one PI species was present in Soy-PI (Fig. 3). Tandem MS of the two highest PI species revealed the presence of PI 16:0/18:2 and PI 18:0/18:2 (see ESM Fig. S6). Additional PIs containing fatty acyl chains with more or less double bonds are also present albeit at lower intensities. We made use of the intensities as well as the theoretical isotope pattern of the observed PI species to relatively quantify their distribution (Pie charts, Fig. 3). Accordingly, PI 34:2 (i.e. PI 16:0/18:2) is the most abundant lipid species in the lower distribution (831–837 *m/z*) while the upper peak distribution (857–865 *m/z*) contains more than one abundant species (Fig. 3).

## Discussion

We introduce a novel sample preparation strategy to analyse phospholipids directly from lipid bilayers. Our strategy involves preparation of liposomes from either standard lipid preparations or natural lipid mixtures. In principle, the workflow described here allows the analysis of every lipid class. However, there are some lipids which poorly hydrate, for instance phosphatidylethanolamine [12], and require special considerations and adjustments. An advantage of the described strategy is, on the other hand, that contaminants which are not hydratable in aqueous buffers will not be included in the lipid sheets during liposome formation and will be excluded from the analysis.

Preparation of liposomes involves several steps and is rather time-consuming; however, we found that extended purification yielding ULVs is not required for simple lipid identification and the use of MLVs is sufficient. However, in the cases where ULVs are needed for preceding experiments, their analysis is also possible allowing for lipid identification in these vesicles.

The use of a high-resolution, high-sensitivity mass spectrometer allows determination of accurate masses of intact lipids including those of low intensity. As some lipid classes yield characteristic fragment ions in either positive or negative ion mode, the ability to analyse lipids in both ion modes is, however, desirable. Therefore, mass spectrometers which can immediately switch between both ion modes are preferred [13]. Fragmentation of selected precursors allows the determination of fatty acyl chains (i.e. length of the hydrocarbon chains and number of double bonds) and unambiguously allows identification of the lipid class. Spectra obtained from MLVs in this study showed high quality even for low abundant precursors.

Importantly, we could show that our strategy allows relative quantification of different lipid species of the same lipid class and therefore enables a quantitative description of the lipid content in natural membranes and tissues. The spontaneous formation of MLVs during hydration in a water-based buffer ensures inclusion of phospholipids into the liposomes. Our strategy therefore provides a real measure of lipid quantities. This is of particular importance for determination of abundant species and the distribution of lipids in unmodified and naturally occurring membranes.

The workflow described here can be further extended by automated analysis during MS data acquisition as well as data analysis employing available software tools [14]. Water-soluble contaminants can potentially be removed from the analysis mixture, for instance by size exclusion chromatography of prepared liposomes. If required, the liposome preparation protocol can be modified according to the needs of preceding or following experiments. We envision that our strategy can be further explored to study natural membrane mimics containing additional membrane and even protein components.

## Electronic supplementary material

ESM 1 (PDF 894 kb)
