# Supplementary material for: A novel sample preparation strategy for shotgun lipidomics of phospholipids employing multilamellar vesicles
Source: Anal Bioanal Chem. 2018 May 8;410(18):4253–8. doi: 10.1007/s00216-018-1113-8 (PMC6021460; doi:10.1007/s00216-018-1113-8)
Supplement: Supplementary file 1 — (PDF 894 kb) [file 216_2018_1113_MOESM1_ESM.pdf]

Analytical and Bioanalytical Chemistry

Electronic Supplementary Material

A novel sample preparation strategy for shotgun lipidomics of phospholipids employing multilamellar vesicles

Melissa Frick, Tommy Hofmann, Caroline Haupt, Carla Schmidt

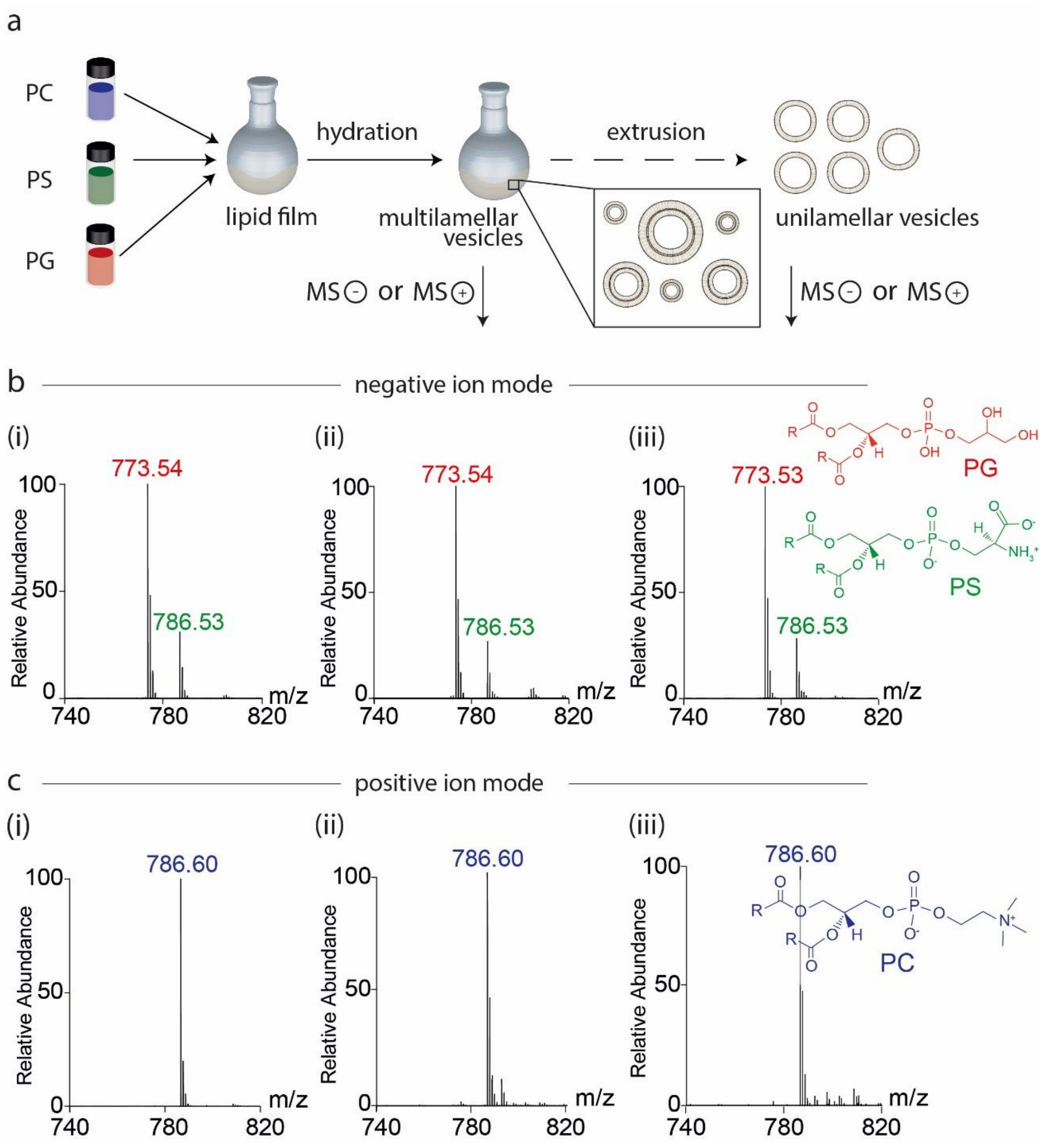

Fig. S1 (a) Workflow of liposome-based MS analysis of lipids. Liposomes were prepared by mixing different lipid proportions of PC (blue), PS (green) and PG (red). The solvent was evaporated by rotary evaporation yielding a lipid film. To obtain MLVs the dry lipid film was hydrated with 200 mM ammonium acetate. ULVs were prepared from MLVs by extrusion through a membrane. (b) MLVs (i), ULVs (ii) and lipids dissolved in organic solvents (iii) were analysed in negative ion mode. PG 18:1/18:1 and PS 18:1/18:1 were observed at *m/z* 773 and 786, respectively. (c) MLVs (i), ULVs (ii) and lipids dissolved in organic solvents (iii) were analysed in positive ion mode. PC 18:1/18:1 was observed at *m/z* 786

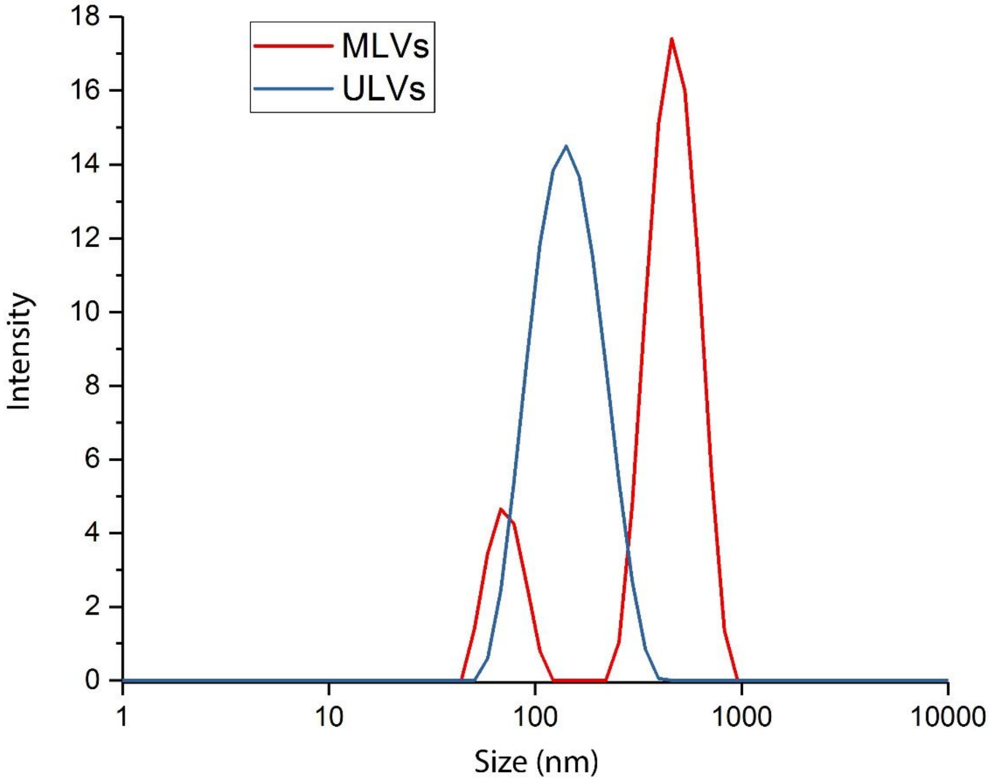

Fig. S2 Dynamic light scattering of MLVs and ULVs. ULVs were prepared by extrusion through a 0.2 µm polycarbonate membrane resulting in a defined average diameter when compared with MLVs

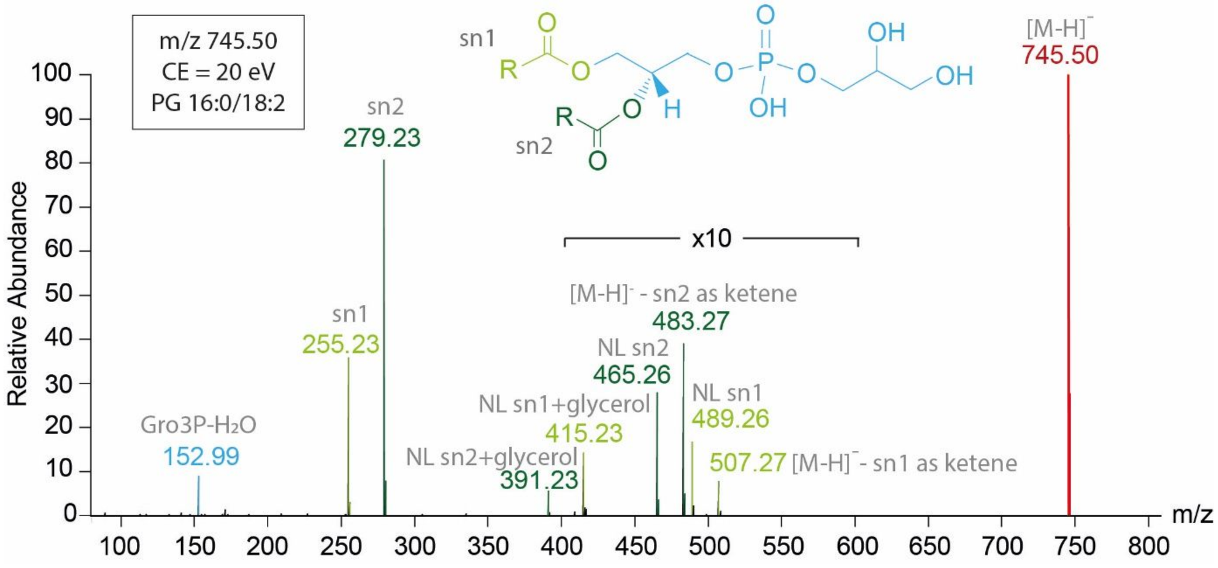

Fig. S3 Tandem mass spectrum of PG 16:0/18:2 (*m/z* 745.50). Specific fragment ions corresponding to the PG head group (cyan) or the sn1 and sn2 fatty acyl chains (light and dark green) were obtained

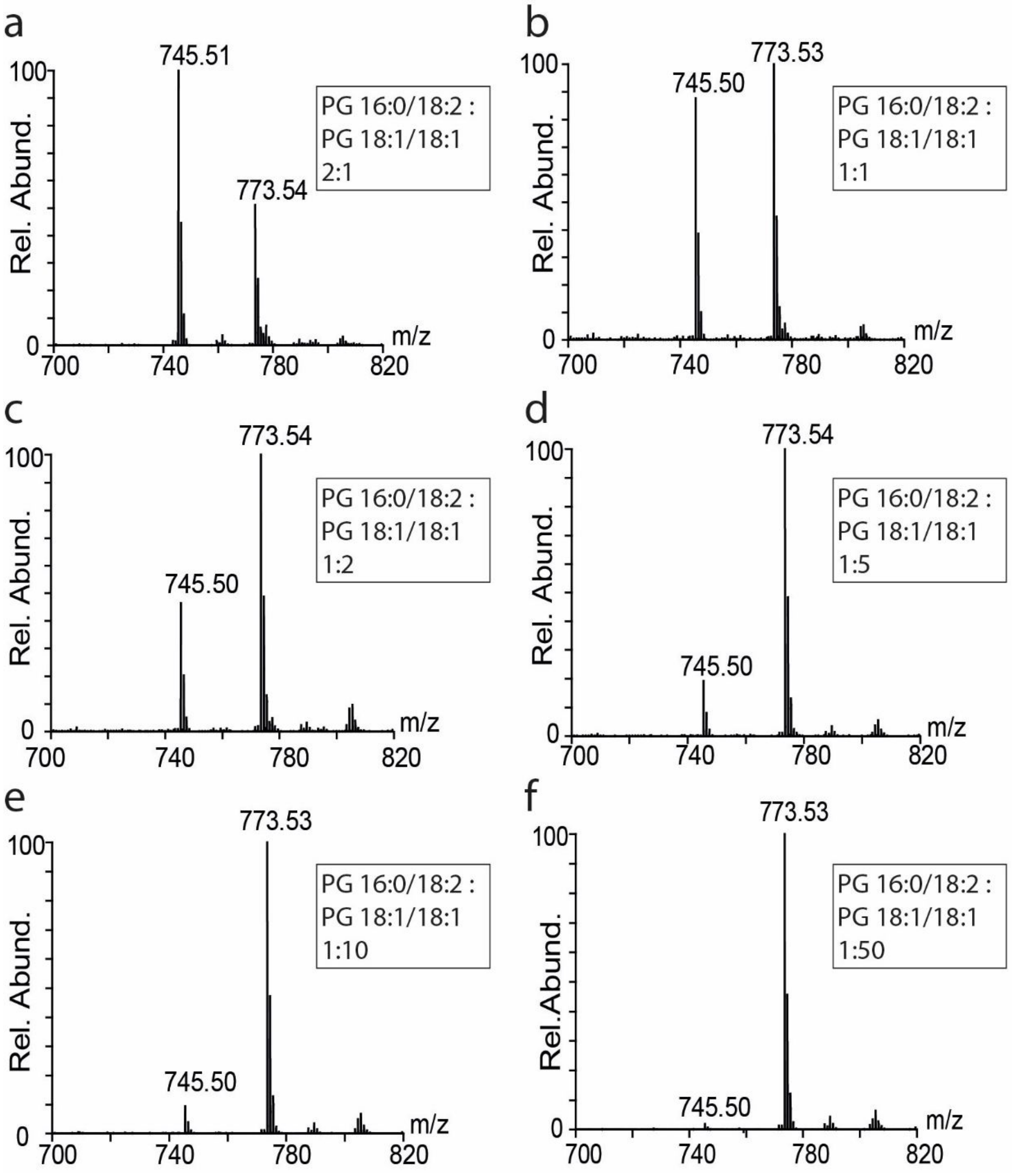

Fig. S4 Relative quantification of PG 16:0/18:2 versus PG 18:1/18:1. PG 16:0/18:2 (*m/z* 745.50) and PG 18:1/18:1 (*m/z* 773.53) were mixed in 2:1 (a), 1:1 (b), 1:2 (c), 1:5 (d), 1:10 (e) and 1:50 (f) ratios

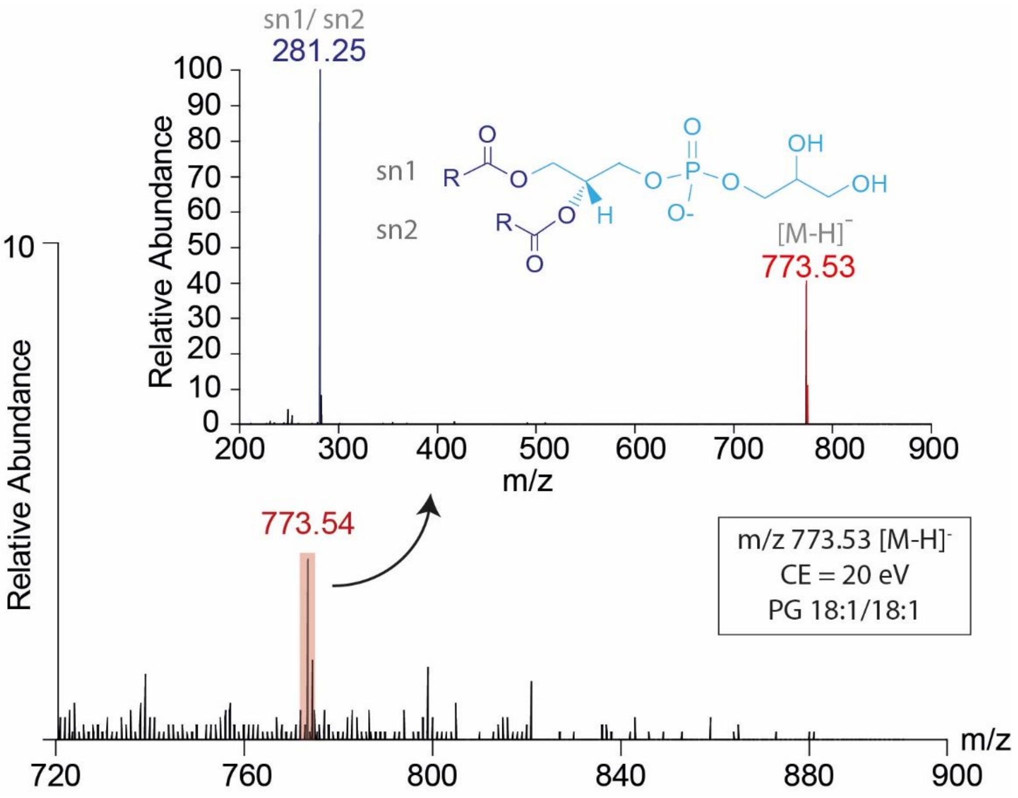

Fig. S5 Identification of PG 18:1/18:1 (m/z 773.54) in liposomes diluted to 500 nM total lipid concentration. Of note, relative abundance is shown at 10 %. Fragment ions specific for the fatty acyl chain were still observed at these low intensities

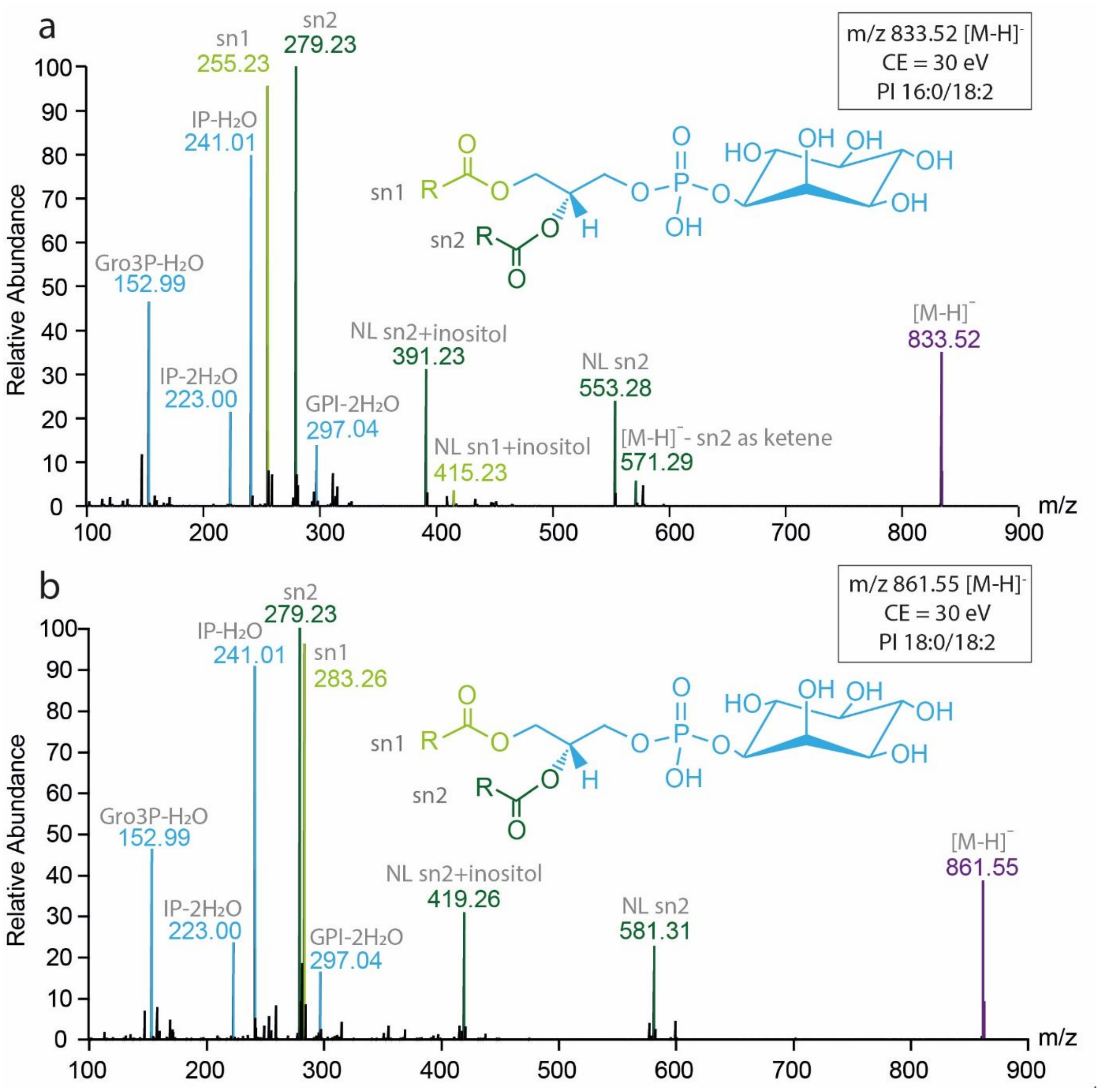

Fig. S6 Tandem mass spectra of PI isomers. (a) PI 16:0/18:2 (*m/z* 833.52) and (b) PI 18:0/18:2 (*m/z* 861.55) were selected for HCD fragmentation. Fragment ions specific to the head group and the fatty acyl chains were obtained. Fragment ions are labelled: NL, neutral loss; Gro3P, glycerol-3-phosphate; IP, inositol phosphate; GPI, glycerol phosphatidyl inositol

Table S1 The absolute intensity for each mixed ratio (PG 16:0/18:2 versus PG 18:1/18:1) is given for three experiments. The average intensity ratios PG 16:0/18:2 *vs.* PG 18:1/18:1 and PG 18:1/18:1 *vs.* PG 16:0/18:2 are given. The standard deviation for each average intensity ratio is listed

Mixed ratio	Intensity PG 16:0/18:2 *m/z* 745.5	Intensity PG 18:1/18:1 *m/z* 773.5	Average intensity ratio PG 16:0/18:2 *vs.* PG 18:1/18:1	Standard deviation	Average intensity ratio PG 18:1/18:1 *vs.* PG 16:0/18:2	Standard deviation
2:1	497602.0	236142.1	1.97	0.36	0.52	0.11
	4053049.4	1804835.2				
	3677591.0	2355709.5				
1:1	13009217.6	10714584.5	0.94	0.24	1.10	0.24
	20498179.1	26426052.1				
	5102865.0	6052876.5				
1:2	2757592.0	5336290.2	0.52	0.09	1.96	0.34
	14828573.5	24177441.9				
	3826486.3	8840603.1				
1:5	2446900.5	10807006.9	0.21	0.03	4.82	0.69
	2439877.5	10784686.9				
	3656557.2	20531446.1				
1:10	3440926.5	26702533.2	0.12	0.02	8.93	2.02
	3196513.6	24843502.4				
	1511534.2	17030206.8				
1:50	787484.1	32479789.2	0.02	0.02	46.33	7.17
	765409.9	33074830.2				
	664958.6	36257802.8				
